# Aqueous humor levels of syndecan-1 and syndecan-4 associated with OCTA metrics in diabetic retinopathy

**DOI:** 10.3389/fmed.2025.1712044

**Published:** 2025-11-20

**Authors:** Jixian Ma, Yue Bian, Yazhou Qin, Yingni Huang, Yuyao Qu, Li Qin, Anming Xie, Qiuping Liu, Xiayu Xu, Jianqin Lei, Jingming Li

**Affiliations:** 1 Department of Ophthalmology, The First Affiliated Hospital of Xi’an Jiaotong University, Xi’an, China; 2 Department of Ophthalmology, Northwest University First Hospital, Xi’an, China; 3 Department of Ophthalmology, The First Affiliated Hospital of University of South China, Hengyang, China; 4 The Key Laboratory of Biomedical Information Engineering of Ministry of Education, School of Life Science and Technology, Xi’an Jiaotong University, Xi’an, China

**Keywords:** syndecan-1, syndecan-4, optical coherence tomography angiography, aqueous humor, diabetic retinopathy

## Abstract

**Purpose:**

Attenuated endothelium glycocalyx had been shown to be related to vascular damage during diabetic retinopathy (DR). Syndecans were pivotal components of retinal glycocalyx, which could be released from the endothelial surface by a highly regulated proteolytic cleavage. Thus, in the present study, we tested for the associations between diabetic retinal vascular injuries, as evidenced by optical coherence tomography angiography (OCTA) metrics, and levels of syndecan-1 and syndecan-4 in the aqueous humor of diabetic patients.

**Methods:**

A prospectively designed cross-sectional study. The study enrolled 14 patients (16 eyes) with DR, 8 patients (10 eyes) with diabetes mellitus (DM) but no DR (NDR), and 16 patients (20 eyes) without DM (NDM). All participants underwent an OCTA scan with a 3 × 3 mm pattern. The images were meticulously binarized for quantification. OCTA metrics of perfusion density (PD) were calculated after large vessels were separated from capillaries by a rigorous automatic algorithm. All vessels PD (PD_all_), large vessels PD (PD_large_), and capillaries PD (PD_cap_) were calculated, respectively. Additionally, the concentrations of syndecans were measured using the highly sensitive Luminex Multiplex Immunoassay, ensuring the accuracy and reliability of the results.

**Results:**

The aqueous humor level of syndecan-1 in DR (298.500 ± 106.900 pg./mL) group was significantly higher than that in NDR (193.800 ± 51.920 pg./mL) and NDM (181.900 ± 42.580 pg./mL) group (both *p* < 0.05). For syndecan-4, the level in aqueous humor in DR (83.270 ± 19.180 pg./mL) group was significantly lower than that in NDR (110.300 ± 13.720 pg./mL) and NDM (116.900 ± 40.570 pg./mL) group (both *p* < 0.05). But the differences of syndecan-1 and syndecan-4 had no statistical significance between NDM and NDR group (both *p* > 0.05). About the OCTA metrics, the PD_all_ in DR (37.840 ± 3.552) group was significantly lower than that in NDR (42.760 ± 2.633) and NDM (46.460 ± 2.201) group (both *p* < 0.05). For the PD_large_, the value in DR (15.940 ± 4.094) group was significantly higher than that in NDR (12.600 ± 3.179) and NDM (11.850 ± 2.565) group (both *p* < 0.05). And the PD_cap_ in DR (21.900 ± 4.581) group was significantly lower than that in NDR (30.150 ± 2.521) and NDM (31.820 ± 2.535) group (both *p* < 0.05). But the differences of PD_all_, PD_large_, and PD_cap_ had no statistical significance between NDM and NDR group (both *p* > 0.05). The results showed that syndecan-1 level in aqueous humor was negatively correlated with PD_all_ (*r* = −0.5499, *p* < 0.0001), PD_cap_ (*r* = −0.4938, *p* = 0.0005). However, syndecan-4 level in aqueous humor was positively correlated with PD_all_ (*r* = 0.3149, *p* = 0.0330), PD_cap_ (*r* = 0.3470, *p* = 0.0181). There were no correlation between syndecan-1 and syndecan-4 with PD_large_ (both *p* > 0.05). ROC analysis showed that syndecan-1 (AUC = 0.8188, *p* = 0.0072), syndecan-4 (AUC = 0.8750, *p* = 0.0016), PD_all_ (AUC = 0.8563, *p* = 0.0027), PD_large_ (AUC = 0.7625, *p* = 0.0269), and PD_cap_ (AUC = 0.9375, *p* = 0.0002) all demonstrated good diagnostic performance for DR. The combination of all indicators for diagnosing DR achieved an AUC of 0.9750, *p* < 0.0001.

**Conclusion:**

The differential expression of syndecan-1 and syndecan-4 was observed in the aqueous humor of DR patients, with syndecan-1 being up-regulated and syndecan-4 down-regulated. As DR progresses, retinal large vessel PD showed an increasing trend, while capillaries PD decreased. Moreover, both syndecan-1 and syndecan-4 exhibited correlations with retinal capillaries PD. Syndecan-1, syndecan-4 and OCTA parameters had demonstrated excellent diagnostic efficacy for DR. These results not only expanded the range of potential biomarkers for detecting DR but also provide support for the future development of novel therapeutic targets for this condition.

## Introduction

Diabetic retinopathy (DR) is one of the common microvascular complications of diabetes mellitus (DM) and the main cause of preventable blindness in the working-age population (1, 2). The International Diabetes Federation estimates that by 2045, the number of DM patients worldwide will reach 700 million and the number of DR patients will increase to 160.5 million (3). More and more evidence indicates that inflammation and angiogenesis are important factors in the occurrence and development of DR, and retinal inflammation persists from the early stage of DR to the terminal stage that endangers vision (4–7). The interaction of multiple biological factors such as inflammatory cytokines, chemokines, adhesion molecules, and vascular endothelial growth factor (VEGF) forms a complex molecular network, which spreads the pathological cascade of inflammation and angiogenesis in DR (6–9). The current treatment methods for DR are mainly used in the advanced stage of the disease. Therefore, it is necessary to seek new targets to identify the initial stage of DR and monitor the effect of applied treatments.

The value of biomarkers in understanding the pathogenesis of DR and developing treatment methods has been well illustrated in the identification of VEGF in intraocular fluid during diabetic macular edema (DME) and proliferative diabetic retinopathy (PDR). This enables us to understand the role of VEGF in retinal leakage, inflammation and angiogenesis, and subsequently anti-VEGF drugs are developed for clinical practice (5). Systemic biomarkers of DM, such as disease duration, poor glycemic control, elevated blood pressure and lipid levels, are related factors, but they cannot determine the deterioration of DR. Therefore, it is crucial to be able to identify the severity and dynamic changes of retinopathy at different stages of DR and link their occurrence to any stage of progression from DR to vision-threatening DR (10, 11). Seeking new biomarkers for the early identification of individuals with an increased risk of microvascular complications in DM may provide better opportunities for the timely implementation of effective therapeutic interventions (5, 12). With the continuous advancement of research on DR, the roles of various biological factors in the vitreous and/or aqueous humor in the pathogenesis, progression, and treatment of the disease have garnered increasing attention. To date, researchers have identified a number of widely recognized biomarkers closely associated with DR, such as inflammatory cytokines, chemokines, adhesion molecules, and vascular growth factors (4). Analysis of the vitreous and/or aqueous humor will enable the characterization of the intraocular phenotype in individuals with DM and aid in identifying those at risk of developing ocular complications or disease progression. Liquid biopsy using ocular fluids will help identify reliable biomarkers, which can be used to monitor drug responses or tailor treatment strategies in a personalized manner (13). In addition, changes in the retinal microvasculature have been demonstrated to occur prior to the appearance of pathological features such as microaneurysms or hemorrhages, providing prognostic value for predicting DR (5). Optical coherence tomography angiography (OCTA), as a novel, non-invasive, three-dimensional imaging modality, can detect changes across retinal layers in DR, assess the effects of various treatments on the retinal microvasculature, and evaluate correlations between functional levels and anatomical and vascular metrics (14). More and more studies indicate that OCTA is capable of quantitatively evaluating DR and its complications, even prior to the onset of clinically visible lesions (14–16). In both clinical applications and DR research, OCTA can identify promising and sensitive biomarkers under various imaging modes, providing new insights into early-stage pathophysiology and supporting therapeutic screening for DR (14).

Hyperglycemia induces vascular dysfunction, inflammation, and neurodegeneration in the retina. Collectively, these processes cause endothelial cell injury and disrupt the blood-retinal barrier (BRB), ultimately leading to the formation of acellular capillaries and edema in the retinal vasculature. The resulting vascular damage promotes ischemia and pathological neovascularization, which exacerbates the severity of DR and can eventually lead to vision impairment (17). The glycocalyx serves as a critical regulator of vascular permeability, and its loss contributes to increased microvascular permeability, leukocyte adhesion, and capillary occlusion, thereby promoting the progression of DR (17–21). Syndecans, a major component of the glycocalyx, belong to the heparan sulfate proteoglycan (HSPG) superfamily. They are transmembrane glycoproteins widely expressed across species and consist of four members (syndecan-1, -2, -3, -4), each exhibiting different functions and expression profiles (22, 23). These proteins play important roles in cell adhesion, migration, as well as growth factor interaction and signaling (22, 24, 25). Syndecans have a short cytoplasmic domain, a single transmembrane domain, and a large extracellular core protein to which glycosaminoglycan chains—primarily heparan sulfate—are attached. The core proteins of syndecans are believed to confer unique binding capabilities and participate in specific protein–protein interactions, thereby determining the functional specificity of various syndecans-dependent biological processes (26, 27). Under physiological conditions, the extracellular domains of syndecans undergo a certain degree of constitutive shedding. However, in response to specific stimuli, this shedding process can be markedly enhanced. The released soluble ectodomains can function as paracrine or autocrine effectors, mediating intercellular signaling and regulatory functions (23, 24, 28). The shedding of syndecans have been demonstrated to regulate numerous pathophysiological processes, including inflammation, tissue repair, and cancer cell proliferation. Tissue injury is accompanied by cellular stress, the accumulation of leukocyte-derived proteases (such as thrombin, plasmin, and elastase), and the release of growth factors, each of which can accelerate syndecans shedding. As a result, shed syndecans ectodomains are detected in inflammatory fluids, where they are believed to maintain the balance of proteolytic activity and growth factor signaling, thereby modulating the inflammatory response (20, 24). The serum levels of shed soluble syndecan-1 and syndecan-4 have been proven to be associated with various diseases, such as sepsis, ischemia–reperfusion injury, multiple types of cancer, pneumonia, and heart failure (24). However, studies on the relationship between syndecans and ocular diseases are rarely reported.

Based on this rationale, we have designed this prospective cross-sectional study to investigate the association between syndecan-1 and syndecan-4 with DR, with the goal of identifying novel biomarkers for the early detection of individuals at increased risk of DR. This may ultimately facilitate the timely implementation of effective therapeutic interventions.

## Materials and methods

### Demographics

This study was a prospectively designed cross-sectional investigation. The study subjects were selected from patients who visited the Department of Ophthalmology at the First Affiliated Hospital of Xi’an Jiaotong University between February 2020 and March 2021. After clinical evaluation, these patients required surgical interventions including phacoemulsification with intraocular lens implantation for age-related cataract, intravitreal pharmacologic injections for DME, or combined phacoemulsification with intraocular lens implantation and intravitreal injection based on their specific conditions. According to the diagnostic criteria established by the American Academy of Ophthalmology (29), the participants were categorized into three groups: non-DM group (NDM), non-DR group (NDR), and DR group (DR), which included both non-proliferative and proliferative stages. Exclusion criteria: (1) Presence of systemic diseases other than type 2 DM, such as nephropathy, ketoacidosis, hyperosmolar coma, or other major systemic disorders; (2) Ocular diseases other than cataracts and DR; (3) Previous history of intraocular injection therapy; (4) Inability to cooperate with ophthalmic examinations; (5) History of ocular trauma. This study was conducted in accordance with the principles of the Declaration of Helsinki and was approved by the Institutional Review Board of the First Affiliated Hospital of Xi’an Jiaotong University. Written informed consent was obtained from all participants prior to their enrollment in the study.

### Ophthalmic examination

All subjects received a comprehensive ophthalmic examination, including the visual acuity, intraocular pressure with a non-contact tonometer, anterior segment, and fundus examination with slit-lamp biomicroscope.

### Image acquisition

**Figure 1:**
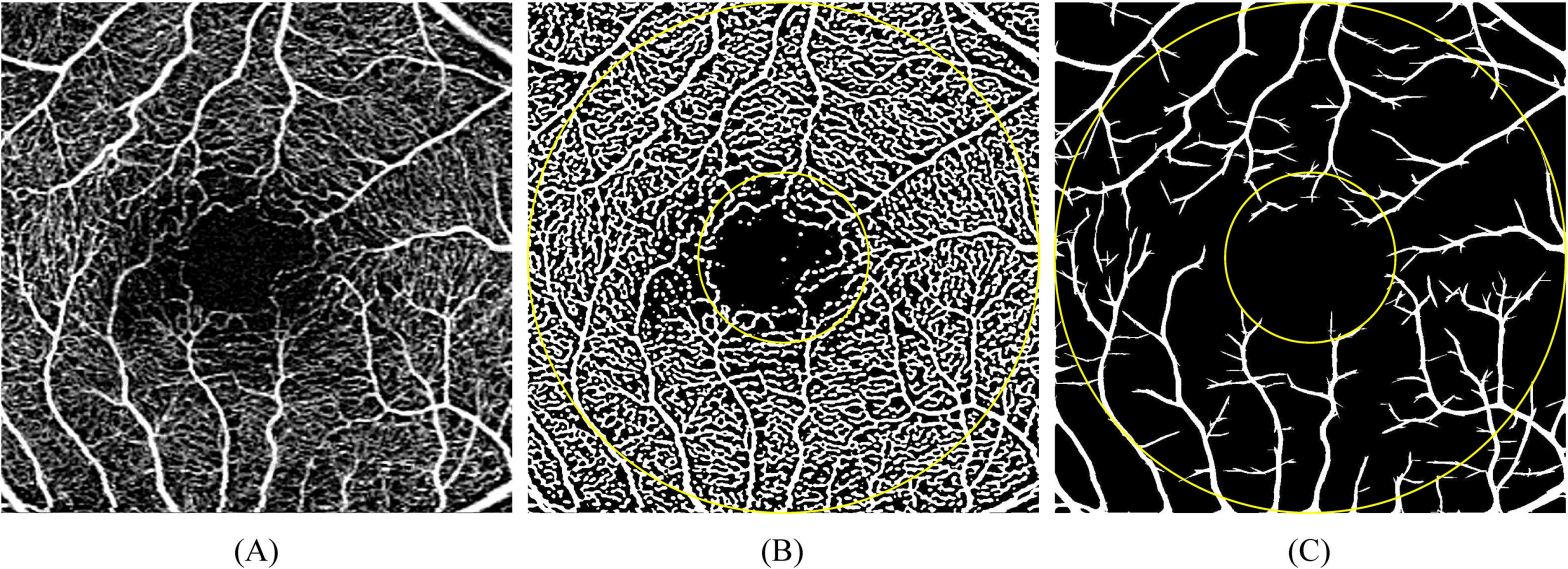
Description of the processing of the en-face images obtained from OCTA (3 × 3 mm scan pattern-1,024 × 1,024 pixels, centered on the fovea). Calculation was performed within the ring between the two yellow circles (the diameter of the inner and outer circle were 1 mm and 3 mm, respectively). **(A)** Original image. **(B)** Binarized image of the superficial vasculature. **(C)** Binarized image of segmented retinal large vessels.

Zeiss OCTA (Cirrus 5000 HD-OCT, Dublin, Germany) was used to obtain the retinal vascular images. En face images of a 3 × 3 mm scan of superficial capillary plexus (SCP, automatically segmented from the internal limiting membrane to the defined boundary of the inner plexiform layer) were obtained by the AngioPlex software (Cirrus 11.1). Perfusion density (PD, defined as the percentage of the vessels within 1 mm-3 mm ring centered on the fovea, based on vascular binarized images) of the retinal all vessels and large vessels were generated separately with the automatic algorithm developed by our group (30). Then, all vessels PD (PD_all_), large vessels PD (PD_large_), and capillaries PD (PD_cap_) were calculated, respectively (Figure 1).

### Collection of aqueous humor and analysis of syndecan-1 and syndecan-4

0.1 mL of aqueous humor was obtained by limbal paracentesis immediately before either phacoemulsification or an intravitreal injection was performed. The aqueous humor was stored at −80 °C immediately after collection with a labeled Eppendorf tube. According to the manufacturer’s protocol, the syndecan-1 and syndecan-4 levels were measured by suspension array technology with Luminex 200 (LXSAHM-13, R&D Systems, Minneapolis, MN, United States). The combined ease of use, high reproducibility, quantification, and sensitivity of multiplex microsphere assay using the Luminex technology make it a valuable technique for everyday performance and an emerging tool for translational and clinical research.

### Statistical analysis

Statistical analysis was performed using GraphPad Prism 10.1.2 (GraphPad, San Diego, CA, United States) and SPSS 22.0 (IBM Corp, Armonk, NY). Measurement data are presented as mean ± standard deviation (SD). The normality of the data was assessed using the Shapiro–Wilk test. Depending on whether the data followed a normal distribution and whether the variances were homogeneous, the following tests were applied: one-way ANOVA with Tukey’s *post hoc* test, Brown-Forsythe test with Dunnett’s post hoc test, or the Kruskal-Wallis test with Dunn’s post hoc test. Spearman correlation analysis was used for non-normally distributed variables. The χ^2^ test was used to compare categorical data. Diagnostic performance was evaluated using receiver operating characteristic (ROC) curve analysis. A *p* value < 0.05 was considered statistically significant.

## Results

### Demographic data

A total of 20 eyes from 16 patients of NDM group, 10 eyes from 8 patients of NDR group, and 16 eyes from 14 patients of DR group were included in the analysis. There were no statistically significant differences in age (*p* = 0.2331), gender (*p* = 0.3109), presence or absence of hypertension (*p* = 0.1365) among the three groups, and the duration of DM (*p* = 0.4216) among the NDR and DR group. Detailed information was shown in Table 1.

**Table 1:** Baseline characteristics of the study subjects in each group.

Characteristics	NDM (*n* = 16)	NDR (*n* = 8)	DR (*n* = 14)	*p*
Age (y)	61.63 ± 12.48	68.38 ± 10.42	59.64 ± 10.84	0.2331^a^
Male gender (n)	11	3	7	0.3109^b^
Hypertension (n)	4	5	3	0.1365^b^
Duration of DM		9.50 ± 6.97	13.15 ± 7.93	0.4216^c^

a One-way ANOVA test.

b Chi-square test-Fisher’s exact test.

c Mann–Whitney test.

NDM, Non-diabetic mellitus; NDR, No-diabetic retinopathy; DR, Diabetic retinopathy; DM, Diabetic mellitus.

### Comparison of syndecan-1 and syndecan-4 levels in aqueous humor

**Figure 2:**
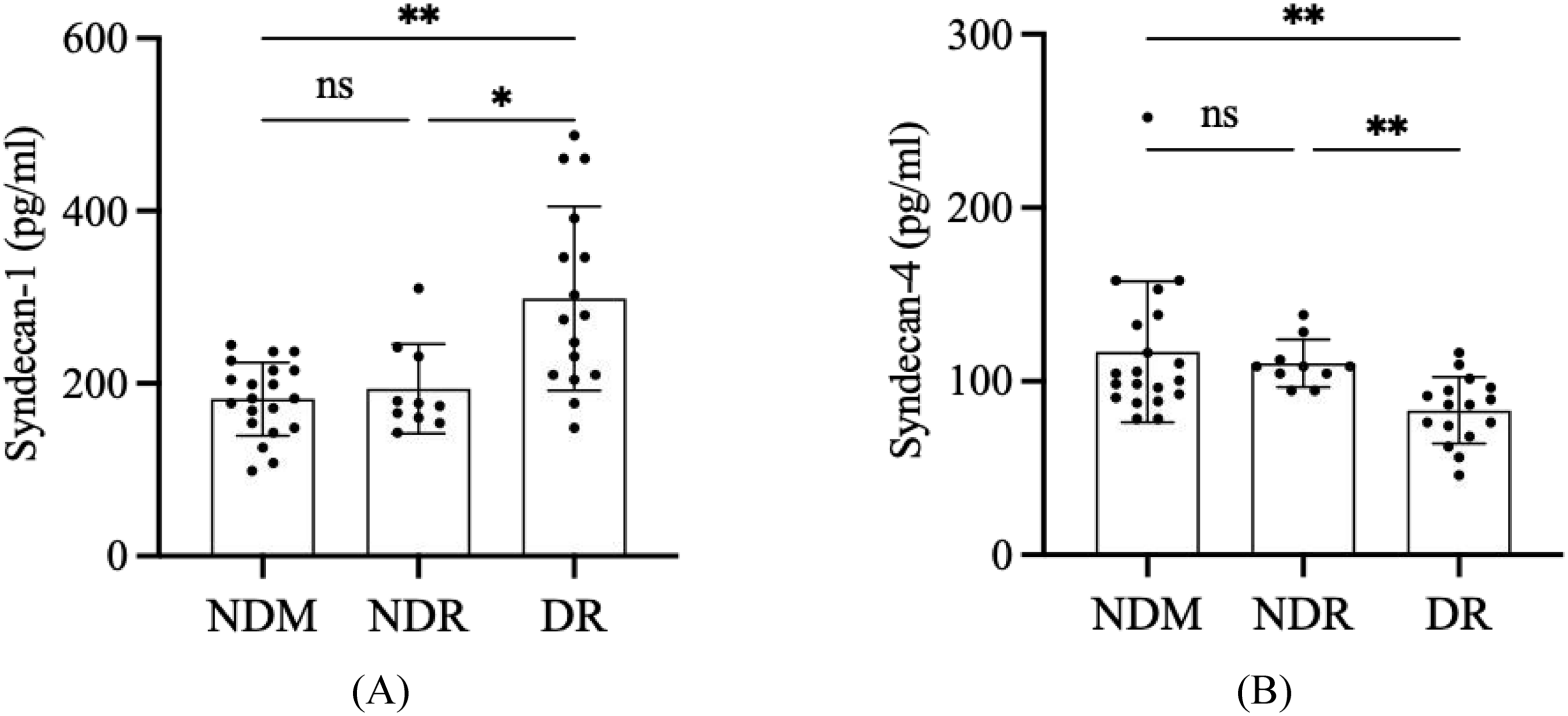
The differential expression of syndecan-1 and syndecan-4 in aqueous humor in NDM, NDR, and DR. The concentration of syndecan-1 **(A)** and syndecan-4 **(B)** in aqueous humor. ns, no significant difference; *p < 0.05; **p < 0.01; ***p < 0.001; ****p < 0.0001. NDM, Non-diabetic mellitus; NDR, No-diabetic retinopathy; DR, Diabetic retinopathy.

The concentrations of syndecan-1 and syndecan-4 in aqueous humor samples of the NDM, NDR, and DR groups were detected by the Luminex liquid suspension chips. The syndecan-1 level in the NDM, NDR, and DR were 181.886 ± 42.577 pg./mL, 193.764 ± 51.917 pg./mL, and 298.521 ± 106.858 pg./mL, respectively. For syndecan-4, the levels in these three groups were 116.939 ± 40.571 pg./mL, 110.269 ± 13.716 pg./mL, and 83.266 ± 19.180 pg./mL, respectively (Figure 2). The differences of syndecan-1 (*p* = 0.0009) and syndecan-4 (*p* = 0.0010) among these three groups were statistically significant. The differences of syndecan-1 (*p* > 0.9999) and syndecan-4 (*p* > 0.9999) had no statistical significance between NDM and NDR. However, there were statistical differences between NDR and DR (*p* = 0.0166 for syndecan-1, *p* = 0.0047 for syndecan-4) and NDM and DR (*p* = 0.0013 for syndecan-1, *p* = 0.0044 for syndecan-4).

### OCTA metrics and the comparison results

**Figure 3:**
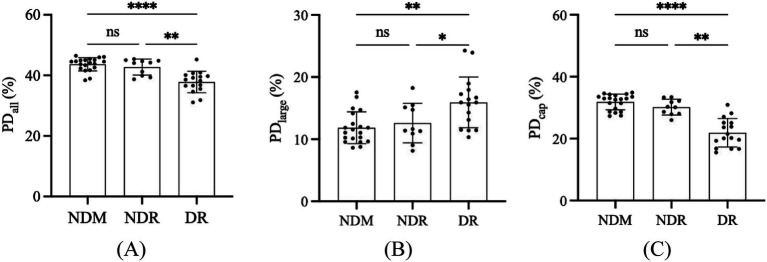
The measured values and comparisons of OCTA metrics among NDM, NDR, and DR. All vessel PD **(A)**, large vessel PD **(B)**, and capillaries PD **(C)**. ns, no significant difference; *p < 0.05; **p < 0.01; ***p < 0.001; ***p < 0.0001. PD, perfusion density; NDM, Non-diabetic mellitus; NDR, No-diabetic retinopathy; DR, Diabetic retinopathy; OCTA, Optical coherence tomography angiography.

In this study, the changes in OCTA metrics were categorized as all vessels PD (PD_all_), large vessels PD (PD_large_), and capillaries PD (PD_cap_). All the OCTA metrics have statistical significance among these three groups, especially between NDM and DR, while there was no significant difference between NDM and NDR. Details were shown in Figure 3. The PD_all_ in the NDM, NDR, and DR were 43.662 ± 2.201, 42.756 ± 2.633 and 37.837 ± 3.548, respectively. For the PD_large_, in these three groups were 11.846 ± 2.565, 12.603 ± 3.179 and 15.942 ± 4.094, respectively. And the PD_cap_ in the NDM, NDR, and DR were 31.817 ± 2.535, 30.153 ± 2.521 and 21.896 ± 4.581, respectively. The differences of PD_all_ (*p* < 0.0001), PD_large_ (*p* = 0.0019) and PD_cap_ (*p* < 0.0001) have significant differences among these three groups. However, the difference between NDM and NDR have no statistical significance in PD_all_ (*p* = 0.5038), PD_large_ (*p* = 0.8247) and PD_cap_ (*p* = 0.6441). Meanwhile, there were statistical differences between NDR and DR (*p* = 0.0037 for PD_all_, *p* = 0.0411 for PD_large_, *p* = 0.0051 for PD_cap_) and NDM and DR (*p* < 0.0001 for PD_all_, *p* = 0.0017 for PD_large_, *p* < 0.0001 for PD_cap_).

### Correlation analysis of syndecan-1 and syndecan-4 levels with OCTA metrics

**Figure 4:**
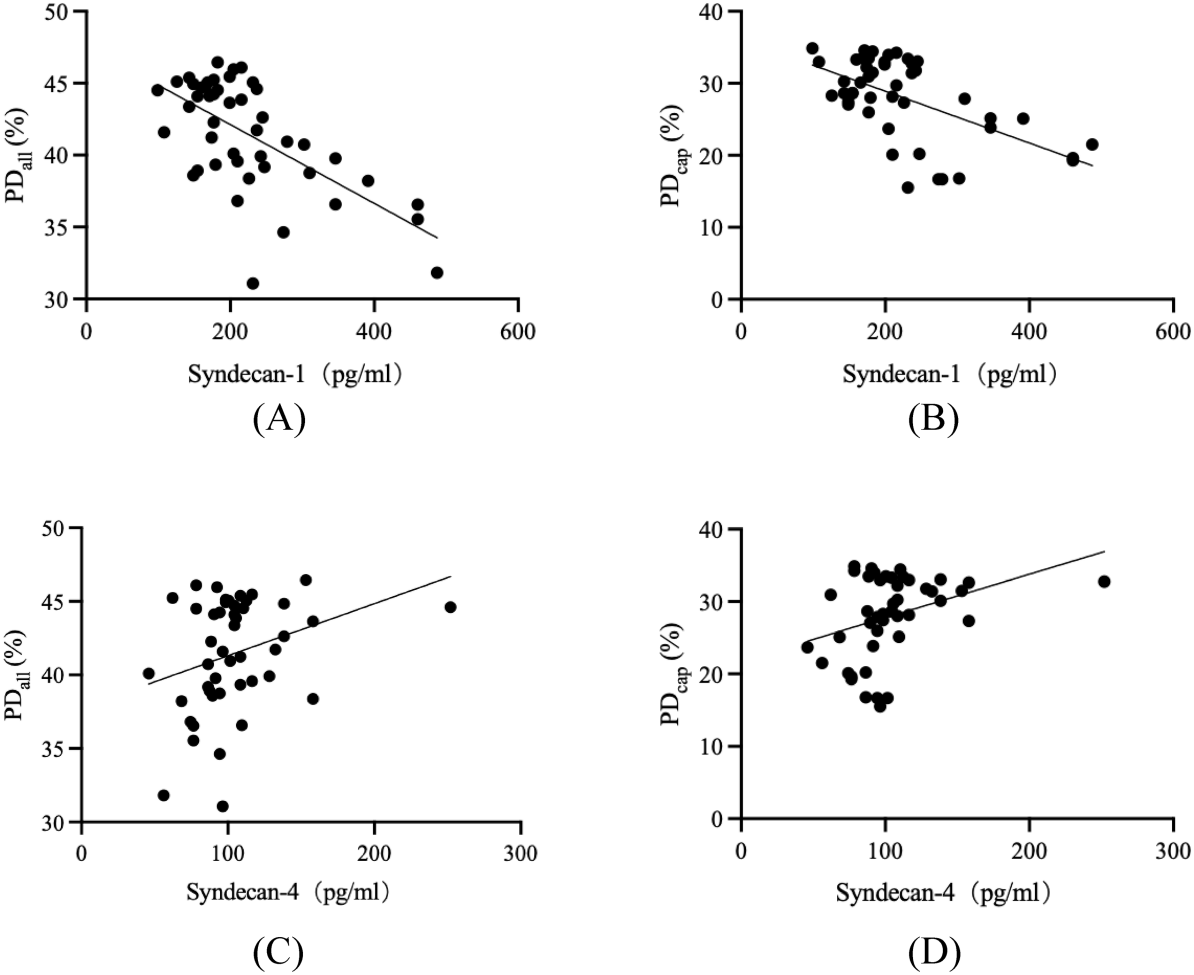
Scatter plots for correlation of aqueous humor syndecan-1 and syndecan-4 with OCTA metrics in NDM, NDR, and DR groups. The correlation of syndecan-1 with PD_all_ **(A)**, and PD_cap_ **(B)**. The correlation of syndecan-4 with PD_all_ **(C)**, and PD_cap_ **(D)**. PD, perfusion density.

Combined each person’s syndecans level in aqueous humor with the OCTA metrics, we found there were some relationships between them as shown in Figure 4. The results showed that syndecan-1 levels were negatively correlated with PD_all_ (*r* = −0.5499, *p* < 0.0001), PD_cap_ (*r* = −0.4938, *p* = 0.0005). The *p* value for syndecan-1 with PD_large_ was 0.2063, with no statistical significance. Compared with syndecan-1, the correlation of syndecan-4 levels in aqueous humor with OCTA metrics does not seem remarkable, syndecan-4 levels in aqueous humor were positively correlated with PD_all_ (*r* = 0.3149, *p* = 0.0330), PD_cap_ (*r* = 0.3470, *p* = 0.0181). The correlation of syndecan-4 with PD_large_ was not significant because *p* value was 0.0596.

### Analysis of the diagnostic efficacy of syndecans and OCTA metrics for DR

**Figure 5:**
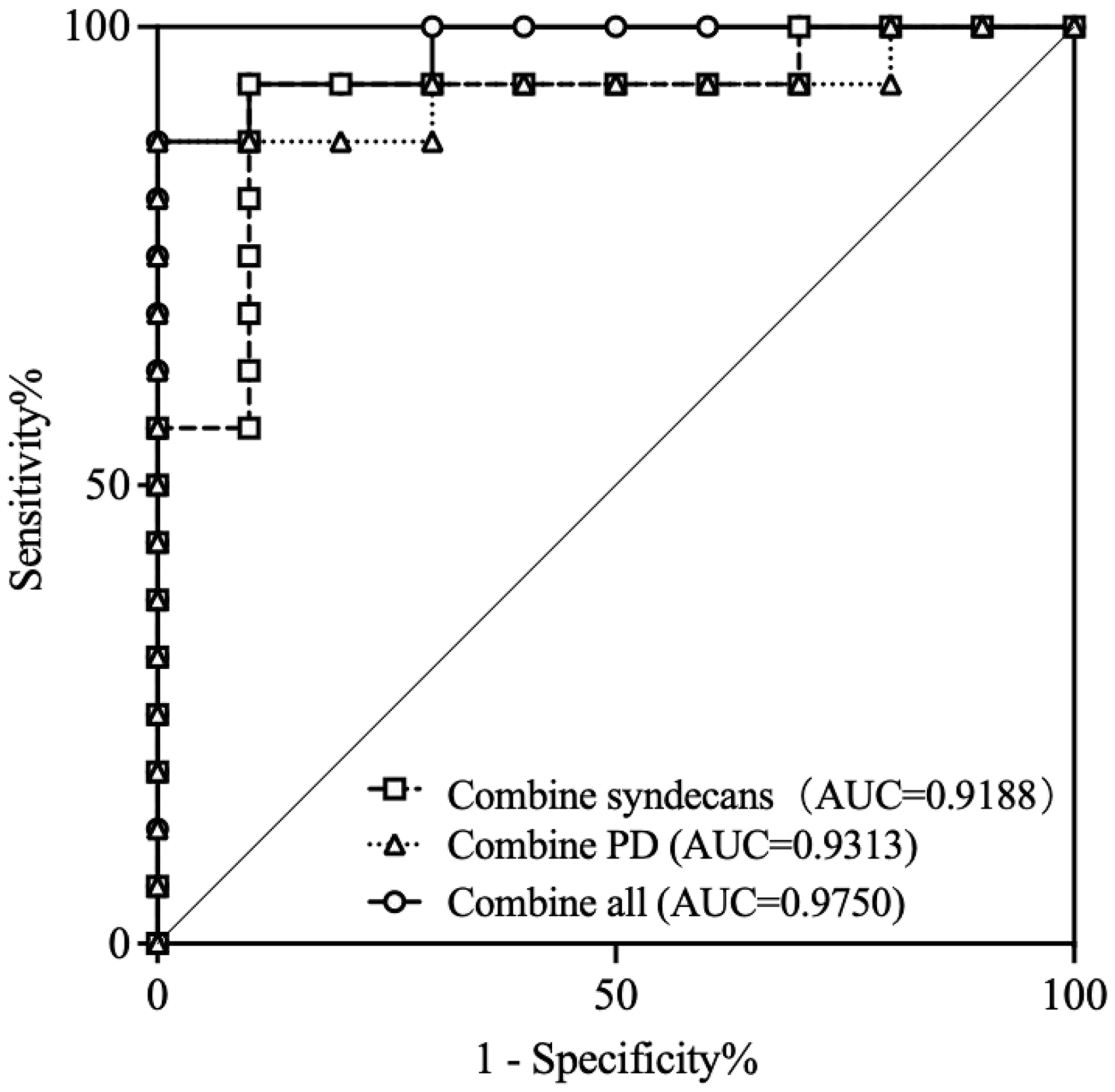
ROC curves for distinguishing DR from DM patients according to combine indicators. ROC, Receiver operator characteristic curve; AUC, Area under the curve; PD, perfusion density; DR, Diabetic retinopathy; DM, Diabetic mellitus.

In further research, the diagnostic efficacy of syndecan-1, syndecan-4, and OCTA parameters for DR in DM patients were analyzed. The results showed that syndecan-1, syndecan-4, PD_all_, PD_large_, and PD_cap_ all demonstrated good diagnostic performance for DR, with all *p* < 0.05. Details were shown in Table 2. Additionally, the combination of syndecan-1 and syndecan-4 for diagnosing DR achieved an AUC of 0.9188 (95% CI, 0.8041 to 1.000), *p* = 0.0004. The combination of PD_all_, PD_large_, and PD_cap_ for diagnosing DR achieved an AUC of 0.9313 (95% CI, 0.8261 to 1.000), *p* = 0.0003. The combination of all indicators for diagnosing DR achieved an AUC of 0.9750 (95% CI, 0.9258 to 1.000), *p* < 0.0001, as shown in Figure 5.

**Table 2:** ROC analysis of syndecans and OCTA metrics in diagnosing DR in DM patients.

Parameters	AUC	AUC 95% CI	*p*	Sensitivity (%)	Specificity (%)	Prediction threshold
Syndecan-1	0.8188	0.6508 to 0.9867	0.0072	87.50	70.00	192.03
Syndecan-4	0.8750	0.7378 to 1.000	0.0016	68.75	100.00	93.00
PD_all_	0.8563	0.7083 to 1.000	0.0027	93.75	70	41.09
PD_large_	0.7625	0.5688 to 0.9562	0.0269	62.5	90	15.53
PD_cap_	0.9375	0.8470 to 1.000	0.0002	81.25	100	25.56

AUC, Area under the curve; PD, perfusion density.

## Discussion

As far as we know, our study was the first to simultaneously report the expression levels of soluble syndecan-1 and syndecan-4 in the aqueous humor of DR patients. Our study utilized Luminex multiplex assay technology to detect the expression levels of syndecan-1 and syndecan-4 in the aqueous humor of the participants. The results showed that the level of syndecan-1 in the aqueous humor of NDR patients was higher than that in NDM patients, but not significantly (*p* > 0.05). And the level of syndecan-1 in the aqueous humor of DR patients was significantly higher than that in both NDR and NDM patients (both *p* < 0.05). Additionally, the level of syndecan-4 in the aqueous humor of NDR patients was lower than that in NDM patients, but not significantly (*p* > 0.05), while the level of syndecan-4 in the aqueous humor of DR patients was significantly lower than that in both NDR and No-DM patients (both *p* < 0.05). These findings suggest that soluble syndecan-1 and syndecan-4 may play distinct roles in the development and progression of DR.

Since the shedding of syndecans was specifically regulated under pathological conditions, the soluble extracellular domains of syndecans were used as biomarkers for various diseases. Syndecans shedding had been shown to modulate numerous pathophysiological processes, such as inflammation, tissue repair, and cancer cell proliferation. Tissue injury was accompanied by cellular stress, accumulation of leukocyte-derived proteases, and release of growth factors, each of which may accelerate syndecans shedding. Consequently, shed syndecans extracellular domains were found in inflammatory fluids and are believed to help maintain the balance of proteolysis and growth factors, as well as mediate inflammation (23–25, 31, 32). The majority of research on soluble syndecans as biomarkers had focused on soluble syndecan-1. For instance, elevated levels of the syndecan-1 extracellular domain had been observed in the serum of patients with sepsis, ischemia–reperfusion injury, graft-versus-host disease, and various cancers (24, 31). In contrast, soluble syndecan-4 in serum was primarily associated with pneumonia and heart failure. Furthermore, high serum levels of syndecan-4 had been identified as a significant predictor of heart failure in hypertensive patients, and syndecan-4 was also considered a suitable biomarker for adverse left ventricular remodeling in patients with dilated cardiomyopathy (24). Serum levels of both soluble syndecan-1 and syndecan-4 had been demonstrated to be associated with a variety of diseases. Consequently, distinguishing the underlying causes of elevated syndecans levels in different patients to determine their potential disease associations remains a challenge. We analyzed the diagnostic efficacy of syndecan-1 and syndecan-4 in the aqueous humor, along with OCTA parameters, for DR. The results demonstrated that syndecan-1, syndecan-4, PDall, PDlarge, and PDcap all exhibited good diagnostic value for DR, with AUC values of 0.8188, 0.8750, 0.8563, 0.7625, and 0.9375, respectively (all *p* < 0.05). Furthermore, the combined diagnostic model integrating these indicators achieved an AUC of 0.9750 (*p* < 0.0001). These findings suggested that a comprehensive evaluation incorporating the expression levels of aqueous humor syndecan-1 and syndecan-4, together with OCTA parameters, holder promise for diagnosing DR. Theoretically, syndecan-1 and syndecan-4 were feasible as diagnostic biomarkers for DR.

Syndecan-1 was the most well-characterized syndecan and seems to be involved in multiple endothelial-related physiological processes (22). As a widely expressed cell surface HSPG, syndecan-1 had been shown to be present in the corneal, limbal, and conjunctival epithelial cells, meibomian and lacrimal glands, as well as in the retina, retinal pigment epithelium, and choroid (33). Soluble (shed) syndecan-1 actively promoted inflammation, angiogenesis, tumor cell growth, and metastasis (24, 28). Kaur et al. (17) founded that soluble syndecan-1 was significantly increased in DM rats and treated rat retinal microvascular endothelial cells (RRMECs) with high glucose. They proposed that this increase could promote pathways related to inflammation, neuronal degradation, and neovascularization, ultimately driving disease progression. Furthermore, Abu El-Asrar et al. (28, 34, 35) reported that the level of soluble syndecan-1 in the vitreous of patients with proliferative diabetic retinopathy (PDR) was significantly higher than that in NDM patients. Their work also highlighted the roles of the heparinase/syndecan-1/VEGF axis and the calcium-binding protein S100A4/syndecan-1/VEGF axis in angiogenesis and progression within the PDR microenvironment. Additionally, Abu El-Asrar et al. (35) demonstrated that intervention with ADAMTS13 (a protease) in high glucose-treated, inflammation-stimulated, and hypoxia-cultured human retinal microvascular endothelial cells (HRMECs) significantly reduced the elevated levels of soluble syndecan-1 in the culture medium. These findings suggest a protective role for ADAMTS13 in retinal endothelial barrier function. Wu et al. (36) observed significantly higher levels of syndecan-1, PIGF, ANGPTL-4, VEGF, and IL-8 in the vitreous of PDR patients compared to NDM patients. They also found that VEGF levels significantly correlated with levels of syndecan-1, PIGF, and ANGPTL-4. The authors proposed that correlations exist among different angiogenic and inflammatory mediators, indicating interactive roles of these pathways in the pathogenesis of PDR. They further suggested that the ANGPTL4/MMP/syndecan-1/IL-8 axis might contribute to PDR progression, providing clues to understanding the link between angiogenesis and inflammation in PDR. Similarly, our previously published research alson had demonstrated the roles of aqueous humor ANGPTL4 and ANGPTL6 in DME (37). Future studies we will continue to explore the role of the ANGPTL4/syndecan-1/IL-8 axis in DR. Currently, reports on soluble syndecan-1 in ocular diseases were scarce. Our study indicated that syndecan-1 may promote inflammation and angiogenesis in the development and progression of DR. This undoubtedly enriched the understanding of the potential roles of soluble syndecan-1 in ocular diseases and provided evidence supporting the development of new therapeutic targets for DR.

Syndecan-4 was nearly ubiquitous and present in most nucleated cell types, though its expression levels were generally low (23). Kaur et al. (17) treated RRMECs with high glucose and observed a significant increase in both syndecan-4 mRNA and protein expression levels. Li et al. (38) founded that the expression of the syndecan-4 extracellular domain decreased in human umbilical vein endothelial cells (HUVECs) treated with advanced glycation end products (AGEs), while the level of soluble syndecan-4 in the culture medium increased. In DM rats, plasma level of soluble syndecan-4 increased during the first week after AGE stimulation but decreased over time. The authors suggested that the initial rise in plasma syndecan-4 might reflect substantial shedding of syndecan-4 at the onset of DM, while the subsequent decline could indicate exhaustion after prolonged duration of the disease. Although soluble plasma syndecan-4 was not solely derived from the endothelium, changes in its concentration may signal underlying pathophysiological alterations. De Rossi et al. (39) demonstrated that retinal angiogenesis during development was unaffected in syndecan-4^−/−^ mice. However, in models of oxygen-induced retinopathy (OIR) and laser-induced choroidal neovascularization (CNV), pathological neovascularization was significantly reduced in the retinas of syndecan-4^−/−^ mice, highlighting the necessity of syndecan-4 in promoting vascular growth in these two neovascular ocular disease models. The authors proposed that, at least in the context of neovascular ocular diseases, syndecan-4 expression positively correlates with newly formed, immature vessels during pathological angiogenesis in both mouse models and human neovascular disorders. In further studies, they evaluated the role of soluble syndecan-4 in anti-angiogenesis and found that it was comparable to Aflibercept in reducing pathological neovascularization, suggesting that soluble syndecan-4 may exert protective effects against aberrant vessel formation. To our knowledge, our study was the first to report that the level of syndecan-4 in the aqueous humor of DR patients was significantly lower than that in NDR and NDM patients. This finding suggested a potential protective role of soluble syndecan-4 against neovascularization, enriching the understanding of its possible functions in ocular diseases. Meanwhile, it provided evidence supporting the development of new therapeutic targets for DR.

In further investigations, we analyzed the correlation between the levels of syndecan-1 and syndecan-4 in the aqueous humor and various OCTA parameters. First, an analysis of all vessels PD revealed that the PD_all_ in NDR patients was lower than that in NDM patients, though not significantly (*p* > 0.05), DR patients exhibited a significantly lower PD_all_ compared to both NDR and NDM patients (all *p* < 0.05). Our findings were consistent with previous studies (40, 41), indicating a declining trend in macular vascular PD_all_ with the progression of DR. Further analysis of large vessels and capillaries PD, showed, (1) PD_large_ in NDR patients was higher than in NDM patients, though not significantly (*p* > 0.05), while DR patients had significantly higher PD_large_ compared to both NDR and NDM patients (all *p* < 0.05); (2) PD_cap_ in NDR patients was lower than in NDM patients, though not significantly (*p* > 0.05), whereas DR patients exhibited significantly lower PD_cap_ compared to both NDR and NDM patients (all *p* < 0.05). Our results were consistent with previous studies (30, 42), demonstrating that with the progression of DR, large vessels PD tended to increase while capillaries PD decreased. Subsequently, we analyzed the correlation between syndecan-1 and syndecan-4 levels and OCTA parameters. The results revealed that syndecan-1 level in the aqueous humor was negatively correlated with PD_all_ (*r* = −0.5499, *p* < 0.0001) and PD_cap_ (*r* = −0.4938, *p* = 0.0005), but showed no correlation with PD_large_ (*p* > 0.05). In contrast, syndecan-4 level was positively correlated with PD_all_ (*r* = 0.3149, *p* = 0.0330) and PD_cap_ (*r* = 0.3470, *p* = 0.0181), with no correlation observed with PD_large_ (*p* > 0.05). Compared to PD_large_, PD_cap_ more accurately reflected changes associated with syndecans, and the correlation between syndecan-1 level with PD_cap_ was stronger. We speculated that the stronger correlation between aqueous humor factors and PD_cap_ may be due to the significantly higher PD_cap_ relative to PD_large_ in the same individual, regardless of the presence of DM or DR. Thus, syndecans level in the aqueous humor demonstrated moderate to strong correlations with PD_cap_. Regarding the stronger correlation between syndecan-1 level and PD_cap_ compared to syndecan-4, we hypothesized that this may be due to inherent differences in the expression patterns of these two syndecans in the retina, leading to variations in their correlations with PD_cap_. In future studies, we will plan to further investigate the underlying reasons for this disparity through cellular and animal experiments.

Our study also had several limitations. First, the relatively small sample size may have contributed to the lack of statistically significant differences in various parameters between the NDM and NDR groups, and the severity of DR was not classified in this study, resulting in the inability to clarify the differences between the different stages of DR. Second, we only analyzed the data within the 3 × 3 mm range of the macular area, resulting in the loss of peripheral retinal information. Finally, whether the differential expression of syndecan-1 and syndecan-4 in the aqueous humor was associated with the duration of DM remained unclear based on our current data. Future studies, particularly those involving animal models, could help elucidate this relationship.

## Conclusion

In summary, our findings demonstrated differential expression of syndecan-1 and syndecan-4 in the aqueous humor of DR patients, with syndecan-1 being up-regulated and syndecan-4 down-regulated. As DR progresses, retinal large vessel PD showed an increasing trend, while capillaries PD decreased. Moreover, both syndecan-1 and syndecan-4 exhibited correlations with retinal capillaries PD. These results not only expanded the range of potential biomarkers for detecting DR but also provide support for the future development of novel therapeutic targets for this condition.

## Data Availability

The datasets presented in this study can be found in online repositories. The names of the repository/repositories and accession number(s) can be found in the article/supplementary material.
